# Serum Levels and *in vitro* CX3CL1 (Fractalkine), CXCL8, and IL-10 Synthesis in Phytohemaglutinin-Stimulated and Non-stimulated Peripheral Blood Mononuclear Cells in Subjects With Schizophrenia

**DOI:** 10.3389/fpsyt.2022.845136

**Published:** 2022-06-17

**Authors:** Jaśmina Arabska, Adam Wysokiński, Ewa Brzezińska-Błaszczyk, Elżbieta Kozłowska

**Affiliations:** ^1^Department of Old Age Psychiatry and Psychotic Disorders, Medical University of Lodz, Lodz, Poland; ^2^Department of Experimental Immunology, Medical University of Lodz, Lodz, Poland

**Keywords:** CXCL8, fractalkine, IL-10, PBMCs, schizophrenia

## Abstract

**Introduction:**

Although schizophrenia is a severe mental illness, whose etiology is still largely unknown, its pathogenesis may be associated with dysregulation of the immune mechanisms. The present study compares the levels of interleukin (IL)-10, interleukin-8 (CXCL8), and fractalkine (CX3CL1) between schizophrenia patients and healthy controls. It also assesses the ability of peripheral peripheral blood mononuclear cells (PBMCs) to produce these cytokines spontaneously and following mitogen-stimulation.

**Materials and Methods:**

A prospective study was performed of 60 adult schizophrenia patients and 32 controls. CXCL8, IL-10, and fractalkine concentrations were measured in serum and supernatants from cultured PBMCs. Anthropometric (BMI, WHR) and body composition measurements were taken using bioimpedance analysis (BIA) and dual-energy X-ray absorptiometry (DXA).

**Results and Conclusion:**

The schizophrenia patients demonstrated significantly higher levels of serum CXCL8 (schizophrenia: 13.4 ± 15.7 pg/mL, control: 6.9 ± 4.2 pg/mL, *p* = 0.001) and lower level of serum fractalkine (schizophrenia: 22.8 ± 9.9 pg/mL, control: 45.4 ± 84.5 pg/mL, *p* = 0.041). Serum IL-10 levels did not significantly differ. No *in vitro* synthesis of fractalkine was observed. Neither unstimulated or PHA-stimulated CXCL8 secretion differed between the two groups (*p* >0.05). The patients not taking mood stabilizers (MS–) demonstrated significantly higher CXCL8 levels than those on mood stabilizers (MS+) (*p* = 0.03) and control (*p* < 0.001). In addition, the MS- sub-group demonstrated significantly lower serum fraktalkine than controls (*p* = 0.009). These effects could be described as pseudo-normalization of CXCL8 and fractalkine in schizophrenia patients taking mood stabilizers.

## Introduction

Schizophrenia is a severe mental illness, marked by hallucinations, delusions, formal thought disorders and cognitive deficits. Although its etiology is still largely unknown, several mechanisms are believed to play a role in its pathophysiology and dysfunctions in glutamatergic, dopaminergic, serotonergic, and gamma-aminobutyric acid signaling are believed to play key roles. However, there is increasing body of evidence showing that dysregulation of the immune mechanisms is also involved in the pathogenesis of schizophrenia, as well (1–3). A wealth of data indicates that patients with schizophrenia demonstrated alteration in both the number and activity of immune cells as well as levels of humoral factors involved in the immune response (4). Some studies have indicated that people with schizophrenia have an elevated mean percentage of B cells (5–7) and that patients with schizophrenia have an elevated number of monocytes, compared to healthy controls (5). Furthermore, studies suggest the reactivity of peripheral blood mononuclear cells (PBMCs) to stimulation may be altered in the course of schizophrenia (8–10). PBMCs are suspected to function as a model for metabolic alterations in schizophrenia (10).

The inflammatory response is transferred between cells by cytokines, which demonstrate varioys secretion types, and anti- and pro-inflammatory effect (11), and alterations in the levels of both anti-inflammatory and pro-inflammatory cytokines have been observed in patients suffering from schizophrenia. Regarding the pro-inflammatory factors, it was found that serum levels of interleukin (IL)-1β, tumor necrosis factor α (TNF-α), IL-6, interferon (IFN)-γ as well as C-reactive protein (CRP) were significantly higher in schizophrenic patients compared with healthy subjects (12–14). One pro-inflammatory interleukin, CXCL8 is known to affect the peripheral immune system and its receptor is expressed on neurons, astrocytes and microglia (15). Although its precise role in the Central Nervous System remains unclear, current research suggest that serum CXCL8 levels are significantly increased in schizophrenia patients in comparison to healthy controls (16, 17). In addition, fractalkine, also known as CX3CL1 is an unusual chemokine which has been recently found to contribute to inflammation processes in the brain (18). It is expressed by neurons, and its receptor CX3CR1 is present exclusively on microglia (19). CX3CL1/CX3CR1 signaling is thought to influence the immunological response through glial activation, which is often present in neurodegenerative disorders (20). It is also known to contribute to various synaptic processes, including synaptic formation, maturation, integration, pruning, and transmission (21). In light of these observations, fraktalkine has recently become a focus of research into schizophrenia.

Research on the anti-inflammatory cytokine IL-10 has yielded more inconsistent results. Some studies indicate that subjects with schizophrenia exhibited a significant decrease in IL-10 levels in comparison to healthy volunteers (22, 23). However, Frayyeh et al. (24) report a significant increase of IL-10 serum levels in schizophrenic patients compared with the healthy controls.

Following on from these findings regarding the role of immune processes in schizophrenia development the present study evaluates the serum levels of anti-inflammatory cytokine IL-10, and pro-inflammatory chemokines CXCL8 and CX3CL1 in schizophrenic patients. It also examines the ability of their PBMCs to product these cytokines *in vitro*, both spontaneous and following stimulation. In addition, bearing in mind that the concentrations of humoral factors can be affected by body composition and metabolic parameters it also investigates the association between cytokine levels and cardiometabolic parameters.

## Materials and Methods

### Subjects

Sixty adult (age 18–60 years) European Caucasian outpatients, diagnosed with paranoid schizophrenia were enrolled into this prospective study. The patients were recruited from psychiatric outpatient clinics in Central Clinical Hospital of Medical University in Lodz (CSK UM Lodz). All patients underwent a structured interview according to the ICD-10 criteria of schizophrenia.

The control group consisted of 32 randomly selected healthy participants (i.e., volunteers without schizophrenia). These healthy volunteers were interviewed with the use of a standard, semi-structured medical interview (based on the ICD-10), routinely used in our hospital. Any healthy participant with self-reported personal or familial psychiatric history, or any previous psychiatric treatment, was excluded from the study.

Any study subject with neurological, endocrinological, or immunological disorders (e.g., allergy, asthma, and AIDS), acute and chronic inflammatory conditions (e.g., pneumonia, rheumatoid arthritis), systemic diseases, and cancer was excluded from the study. All subjects had normal laboratory tests [including complete blood count, white blood cell (WBC) count, C-reactive protein (CRP), alanine aminotransferase (ALT), aspartate aminotransferase (AST), bilirubin, urea, creatinine, and electrolytes]. All subjects have been informed about the aims and methods of the study and had expressed their written informed consent for participation in this study. The study protocol was approved by the Bioethics Commission of the Medical University of Lodz (RNN/122/16/KE).

### Clinical Assessments

The clinical symptoms of schizophrenia were assessed using the Positive and Negative Syndrome Scale (PANSS) and its sub-scores (positive, negative, and general symptoms), while the severity of depressive symptoms was measured using the Calgary Depression Scale for Schizophrenia (CDSS) (25). The cut-off score for depression in CDSS was >6. For each patient assessments with all scales were performed by one trained rater. The information on medications used by schizophrenia patients was also collected and considered in the analysis.

### Blood Collection and Isolation of PBMCs

Blood samples were collected between 8 and 9 am, after at least an 8-h overnight fasting. For serum tests the samples were collected directly into serum separator tubes and centrifuged (10 min, 3,500 rpm). Serum was collected and stored at −80°C for up to 6 months until assayed.

PBMCs were isolated from whole blood by density gradient centrifugation using Histopaque-1077 (Sigma-Aldrich, Germany). After centrifugation at 400 g for 30 min at room temperature, the buffy coat was aspirated, transferred into a clean conical centrifuge tube, and washed three times in 1x phosphate buffered saline (Sigma-Aldrich, Germany). The viability and concentration of isolated PBMCs are traditionally measured by manual counting with trypan blue using a Bürker chamber.

### Cell Culture

The PBMCs were suspended at a concentration of 10^6^ cells per mL in RPMI-1640 supplemented with 1% penicillin/streptomycin (Sigma-Aldrich, Germany), 2 mM L-glutamate (Gibco, USA), and 10% heat-inactivated fetal bovine serum (Gibco, USA) in 48-well sterile (non-pyrogenic) polystyrene flat bottom plates (Corning, USA) in the presence or absence of 5 μg/mL phytohemaglutinin (PHA; Sigma-Aldrich, Germany). Cells were cultured at 37°C in humidified atmosphere of 5% CO_2_. The cell-free supernatants were collected 72 h later and frozen in aliquots at −80°C.

### Cytokines Measurements and Other Laboratory Tests

CXCL8, IL-10, and fractalkine concentrations in both the serum and supernatants from cultured PBMCs were measured using the Cytometric Bead Array kits (BD Biosciences, USA) according to the manufacturer's protocol. The samples were analyzed with a Calibur flow cytometer (BD Biosciences, USA) using BD FCAP Array Software (BD Biosciences, USA). Results are expressed as pg/mL. Results are expressed as pg/mL. The levels of triglycerides, cholesterol, LDL, HDL, glucose, CRP, aminotransferases (AST, ALT) were measured using an automatic Dirui CS-400 analyser (Dirui, China).

### Anthropometry

Height was measured with a wall-mounted height measure to the nearest 0.5 cm. Weight was measured with a seca 955 (seca, UK) digital chair scale that was kept on a firm horizontal surface, with subjects undressed. Waist and hip circumferences were measured using a non-stretchable fiber measuring tape. Body mass index (BMI) was calculated as body weight in kilogram divided by the height in meter squared (kg/m^2^). Waist-to-hip ratio (WHR) was calculated as waist circumference divided by hip circumference.

### Body Composition

Body composition was measured using two methods: bioimpedance analysis (BIA) and dual-energy X-ray absorptiometry (DXA). BIA was performed using a Maltron BIOSCAN 920-2-S Body Fat Analyzer (Maltron, UK), multi-frequency (5, 50, 100, and 200 kHz) bioelectrical impedance analyser. DXA was performed with a Lunar iDXA scanner (GE Healthcare, UK). The standard operating conditions, including preparation of the participants, electrodes placement and measurement procedures, were monitored by a trained operator. The measurement using DXA and BIA was taken immediately prior to anthropometry measurements with the participants lying supine, resting. Briefly, BIA determines the electrical impedance, or opposition to the flow of an electric current through body tissues which can then be used estimate the total body water content, and thus fat-free body mass and, body fat. In DXA two X-ray beams, with different energy levels, are aimed at the patient different tissue types (bone, muscle, fat) can be determined from the absorption of each beam by tissues.

The following body composition parameters were measured using DXA method: TBF (total body fat), LBM (lean body mass), VAT (visceral adipose tissue) mass, VAT volume. TBF and LBM are expressed both in grams and as percentage of total body mass. SAT (subcutaneous adipose tissue) and VAT areas are measured at the level of the umbilicus, using data input from Maltron BIA analyzer (with special electrodes placement for these measurements) and Maltron software, which converts raw data (impedance and phase angle). Fat mass index (FMI) was calculated as TBF in kilogram divided by the height in meter squared (kg/m^2^).

### Statistical Analysis

Statistical procedures were performed with STATA 15.1 (StataCorp, College Station, USA) and GraphPad Prism 7.00 (GraphPad Software, La Jolla California, USA). Simple descriptive statistics [mean ± standard deviation and 95% confidence interval (CI)] were generated for continuous variables. For discrete variables absolute and relative numbers are presented together with corresponding 95% CI. The normality of distribution was tested by histogram evaluation and the Shapiro-Wilk test. All cytokines concentrations were highly skewed and were transformed using inverse square root (serum CXCL8), log (CXCL8 non-stimulated and PHA-stimulated, serum IL-10, fractalkine) and square root (IL-10 non-stimulated and PHA-stimulated). The transformed serum IL-10, serum fractalkine, CXCL8, and IL-10 PHA-stimulated variables still did not demonstrate a normal distribution and were therefore analyzed using non-parametric tests (Wilcoxon rank-sum test; the *z*-value was reported in tables). Other variables were analyzed using two sample or paired *t*-test (with a correction for unequal variations when necessary; the t value was reported in Tables). Multiple inter-group comparisons were analyzed using analysis of variance (ANOVA) with *post-hoc* Bonferroni multiple-comparison test or Dunn's Pairwise Comparison test for non-parametric variables (the rho value was reported in Tables). Differences in proportions were analyzed by Chi-squared or Fisher's exact test. Associations were tested by Spearman's rank correlation coefficients (with Bonferroni-adjusted significance level). Adjusted means were calculated from linear regression estimates. The significance level was set at *p* < 0.05 (two sided).

## Results

### Demographic and Clinical Information

Detailed demographic and cardio-metabolic parameters are shown in Table 1. Some differences were observed between the two study groups [cigarette smoking, comorbidity of diabetes, WHR, total fat (%), VAT mass (%), total fat-free mass (%), and FMI]. All these variables were later included in adjusted means models.

**Table 1:** Summary of clinical data.

	**Schizophrenia** **(*n* = 60)**	**Control** **(*n* = 32)**	
Men	38 (63.3%) [0.49–0.75]	21 (65.6%) [0.46–0.81]	chi2 = 0.05 *p* = 0.83
Age [y]	39.4 ± 10.4 [36.7–42.1]	37.3 ± 10.3 [33.6–41.0]	*t* = −0.93 *p* = 0.35
Cigarette smoking	37 (61.7%) [0.48–0.73]	5 (15.6%) [0.05–0.33]	**chi2** **=** **17.83** ***p*** **<** **0.001**
Smoking [pack-years]	9.6–12.2 [6.5–12.8]	2.5 ± 6.5 [0.1–4.8]	*z* = −4.25 *p* < 0.001
**Comorbidities**			
Hypertension	11 (18.3%) [0.09–0.30]	7 (21.9%) [0.09–0.40]	chi2 = 0.16 *p* = 0.68
Diabetes	7 (11.7%) [0.05–0.23]	0 (0%) [0–0.11^*^]	**chi2** **=** **4.04** ***p*** **=** **0.04**
Dyslipidemia	18 (30.0%) [0.19–0.43]	6 (18.8%) [0.07–0.36]	chi2 = 1.37 *p* = 0.24
**Anthropometric**			
Weight [kg]	82.3 ± 18.2 [77.6–87.0]	80.5 ± 18.8 [73.8-87.3]	*t* = −0.45 *p* = 0.65
BMI [kg/m^2^]	28.2 ± 5.9 [26.7-29.8]	26.0 ± 5.4 [24.0-27.9]	*t* = −1.82 *p* = 0.07
WHR	0.96 ± 0.08 [0.93–0.97]	0.87 ± 0.09 [0.84–0.91]	***t*** **=** **−4.44** ***p*** **<** **0.001**
**Blood pressure [mm Hg]**			
systolic	125.0 ± 15.6 [121.0-129.1]	131.3 ± 17.5 [125.0-137.6]	*t* = 1.70 *p* = 0.09
diastolic	79.8 ± 10.9 [77.0-82.6]	83.1 ± 10.8 [79.2-87.0]	*t* = 1.38 *p* = 0.17
**Laboratory tests**			
ALT [U/L]	31.4 ± 22.2 [25.6–37.1]	28.5 ± 7.6 [25.8–31.2]	*t* = −0.90 *p* = 0.37
AST [U/L]	25.4 ± 11.4 [22.5–28.4]	30.0 ± 17.7 [23.7–36.4]	*t* = 1.33 *p* = 0.19
Glucose [mg/dL]	94.0 ± 24.5 [87.7–100.4]	87.9 ± 14.5 [82.7–93.2]	*t* = −1.49 *p* = 0.14
CRP [mg/L]	2.8 ± 3.2 [1.9–3.6]	3.8 ± 8.2 [0.8–6.7]	*t* = 0.64 *p* = 0.52
Total cholesterol [mg/dL]	192.6 ± 36.2 [183.3–202.0]	210.6 ± 43.4 [194.9–226.2]	*t* = 1.99 *p* = 0.05
Triglycerides [mg/dL]	136.1 ± 51.8 [122.7–149.5]	178.1 ± 126.9 [132.4–223.9]	*t* = 1.79 *p* = 0.08
HDL cholesterol [mg/dL]	47.9 ± 14.0 [44.3–51.6]	53.2 ± 13.1 [48.4–57.9]	*t* = 1.76 *p* = 0.08
LDL cholesterol [mg/dL]	115.7 ± 32.1 [107.4–124.0]	121.9 ± 36.9 [108.7–135.3]	*t* = 0.81 *p* = 0.42
**Body composition**			
Total fat [g]	29,524.9 ± 12,096.7 [26,372.4–32,677.3]	24,792.8 ± 11,772.2 [20,396.9–29,188.6]	*t* = −1.76 *p* = 0.08
Total fat–free mass [g]	51,099.1 ± 9,507.3 [48,621.5–53,576.7]	51,498.6 ± 10,673.4 [47,513.1–55,484.2]	*t* = 0.17 *p* = 0.86
Total fat [%]	34.8 ± 8.5 [32.6–37.0]	30.3 ± 8.8 [27.0–33.6]	***t*** **=** **−2.35** ***p*** **=** **0.02**
Total fat–free mass [%]	62.9 ± 9.2 [60.5–65.3]	65.9 ± 8.4 [62.7–69.0]	*t* = 1.54 *p* = 0.13
FMI [kg/m^2^]	10.2 ± 4.3 [9.0–11.3]	8.1 ± 4.0 [6.6–9.6]	***t*** **=** **−2.21** ***p*** **=** **0.03**
SAT area [cm^2^]	176.6 ± 81.5 [155.5–197.6]	146.2 ± 68.1 [121.7–170.8]	*t* = −1.89 *p* = 0.06
VAT area [cm^2^]	147.5 ± 81.1 [126.5–168.5]	105.1 ± 67.9 [80.5–129.6]	***t*** **=** **−2.66** ***p*** **=** **0.009**
VAT mass [g]	1,391.6 ± 956.4 [1,137.9–1,645.4]	996.7 ± 1,010.9 [619.2–1,374.2]	*t* = −1.76 *p* = 0.08
VAT mass [%]	1.6 ± 0.9 [1.4–1.8]	1.1 ± 0.9 [0.7–1.4]	***t*** **=** **−2.52** ***p*** **=** **0.01**
VAT volume [cm^3^]	1,475.2 ± 1,013.8 [1,206.2–1,744.2]	1,056.5 ± 1,071.6 [656.3–1,456.6]	*t* = −1.76 *p* = 0.08

* *One–sided, 97.5% confidence interval*.

*Data given as mean ± standard deviation [95% CI], otherwise as n (%) [95% CI]. Bold values are statistically significant*.

Clinically, all study patients were in a stable condition. Clinical aspects of schizophrenia patients and detailed information on psychotropic medication are shown in Tables 2, 3. Their mean treatment duration for schizophrenia was 15.4 ± 10.5 [12.6–18.1] years, number of schizophrenia episodes 6.7 ± 6.2 [5.1–8.3], number of hospitalisations 9.8 ± 11.1 [7.0–12.7], and time from last hospitalization 10.7 ± 16.3 [2.1–2.7] months. All study patients were on antipsychotic pharmacotherapy, with a defined daily dose of 2.4 ± 1.1 [2.1–2.7], with a chlorpromazine equivalent of 733.4 ± 333.5 [647.2–819.5], which indicates at least standard doses. The majority of study patients were taking second-generation antipsychotics [57, 95.0% (0.86–0.99)]. Twenty-two were taking first-generation antipsychotics (36.7%) [0.25–0.50]. Forty (66.7%) [0.53–0.78] were taking more than one medication. Also, 25 (41.7%) [0.29–0.55] patients were taking mood stabilizers (mostly lamotrigine *n* = 11 and valproate *n* = 11) while and 8 (13.3%) [0.06–0.25] were taking antidepressants.

**Table 2:** Clinical aspects of schizophrenia patients.

Mean treatment duration (years)	15.4 ± 10.5 [12.6–18.1]
Number of schizophrenia episodes	6.7 ± 6.2 [5.1–8.3]
Number of hospitalisations	9.8 ± 11.1 [7.0–12.7]
Time from last hospitalization (months)	10.7 ± 16.3 [2.1–2.7]
Defined daily dose	2.4 ± 1.1 [2.1–2.7]
Chlorpromazine equivalent	733.4 ± 333.5 [647.2–819.5]
PANSS score	67.7 ± 15.0 [63.8–71.6]
PANSS P	15.7 ± 5.1 [14.4–17.1]
PANSS N	19.3 ± 4.9 [18.0–20.5]
PANSS G	32.8 ± 7.3 [30.9–34.7]
CDSS score	3.1 ± 3.5 [2.2–4.0]

*Data given as mean ± standard deviation [95% CI]*.

**Table 3:** Psychotropic medications in the study group.

**Second-generation antipsychotics**	**57 (95.0%) [0.86–0.99]**
**First-generation antipsychotics**	**22 (36.7%) [0.25–0.50]**
**>1 medication**	**40 (66.7%) [0.53–0.78]**
**Mood stabilizers**	**25 (41.7%) [0.29–0.55]**
** Lamotrigine**	**11 (18.3%)**
** Valproate**	**11 (18.3%)**
**Antidepressants**	**8 (13.3%) [0.06–0.25]**

*Data given as n (%) [95% CI]*.

The mean severity of schizophrenia symptoms, assessed using the PANSS score, was 67.7 ± 15.0 [63.8–71.6]. The following scores were obtained for the subscales: PANSS P (positive symptoms): 15.7 ± 5.1 [14.4–17.1], PANSS N (negative symptoms): 19.3 ± 4.9 [18.0–20.5], and PANSS G (general symptoms): 32.8 ± 7.3 [30.9–34.7]. More subjects demonstrated dominating negative symptoms [PANSS N > PANSS P: *n* = 44 (73.3%) (0.60–0.84) vs. PANSS P > PANSS N: *n* = 16 (26.6%) (0.16–0.40)]. Severity of depressive symptoms was low [CDSS score 3.1 ± 3.5 (2.2–4.0)], eight (13.3%) [0.06–0.24] patients were classified as depressed using CDSS cut-off core (>6).

### Serum and *in-vitro* Levels

**Figure 1:**
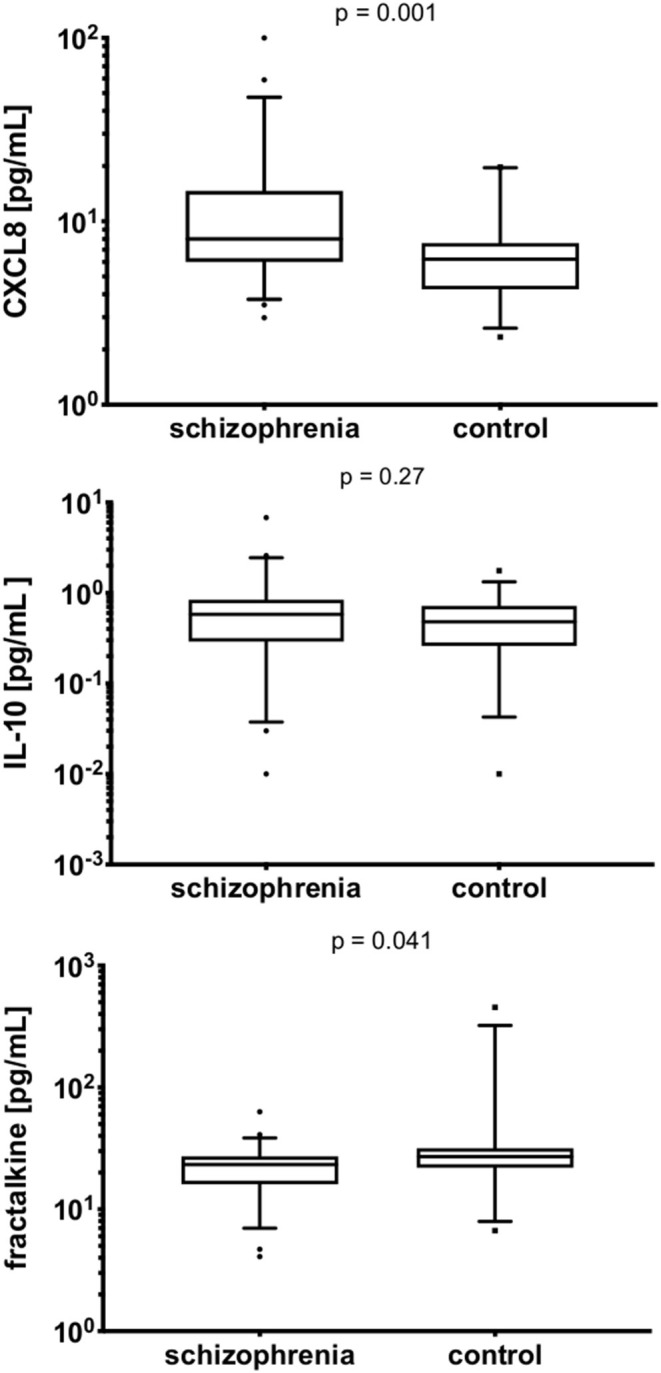
The comparison of serum CXCL8, IL-10, and fractalkine in the schizophrenia and control groups. Y axes are log10 scaled. The boxes extend from 25 to 75th percentiles, the lines in the middle of the boxes represent medians. The whiskers extend from 10 to 90th percentiles.

**Figure 2:**
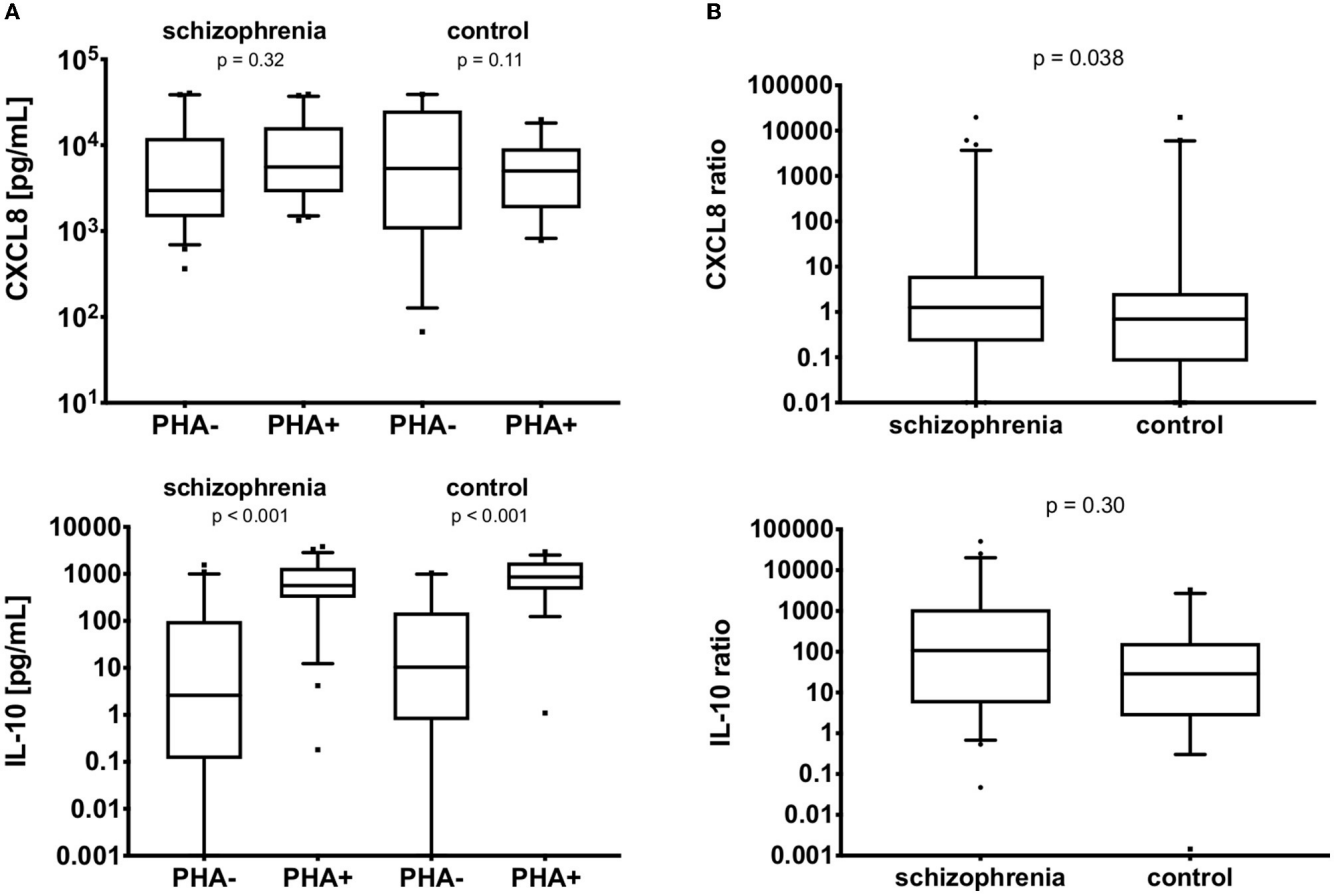
**(A)** *In vitro* CXCL8 and IL-10 production in non-PHA (PHA–) and PHA-stimulated (PHA+) PBMCs. Vertical lines represent 95% CI, black boxes represent means. **(B)** Ratios of *in vitro* CXCL8 and IL-10 production in non-PHA (PHA–) and PHA-stimulated (PHA+) PBMCs. The boxes extend from 25 to 75th percentiles, the lines in the middle of the boxes represent medians. The whiskers extend from 10 to 90th percentiles.

The serum levels of CXCL8, IL-10, and fractalkine are detailed in Table 4. The patients with schizophrenia demonstrated higher levels of CXCL8 (*p* = 0.001) and lower levels of fractalkine (*p* = 0.041; see Figure 1). It was not possible to measure *in vitro* synthesis of fracktalkine in 51 non-stimulated cells and 48 stimulated cells in schizophrenia group, 27 non-stimulated cells and 26 stimulated cells in the control group. Details on the CXCL8 and IL-10 synthesis by PBMCs, both the non-stimulated and PHA-stimulated cells, is shown in Table 4 and Figure 2A. CXCL8 secretion did not differ between the schizophrenia or control group, irrespective of stimulation status (*p* >0.05), while IL-10 secretion was significantly higher in PHA-stimulated PBMCs compared with non-stimulated cells, both in the schizophrenia and control groups (*p* < 0.001). PHA-stimulation induced significantly greater CXCL8 secretion i.e., ratio of PHA-stimulated to non-stimulated secretion in the schizophrenia group (*p* = 0.038), but no significant gain in IL-10 secretion (*p* = 0.30), see Figure 2B.

**Table 4:** Serum and *in vitro* concentrations of CXCL8, IL−10, and fractalkine in the study groups.

	**Schizophrenia** **(*n* = 60)**	**Control (*n* = 32)**	
CXCL8 serum [pg/mL]	13.4 ± 15.7 [9.2–17.5]	6.9 ± 4.2 [5.4–8.5]	***t*** **=** **3.28** ***p*** **=** **0.001**
IL−10 serum [pg/mL]	0.76 ± 0.97 [0.49–1.03]	0.52 ± 0.36 [0.39–0.65]	*z* = −1.10 *p* = 0.27
fractalkine serum [pg/mL]	22.8 ± 9.9 [20.0–25.5]	45.4 ± 84.5 [12.7–78.2]	***z*** **=** **2.05** ***p*** **=** **0.041**
CXCL8 non–stimulated [pg/mL]	10,107.8 ± 13,313.7 [6,438.1–13,777.5]	12,442.9 ± 14,390.4 [7,164.5–17,721.4]	*z* = −0.01 *p* = 0.99
CXCL8 PHA–stimulated [pg/mL]	1,0121.2 ± 1,0211.4 [7,306.6–12,935.8]	6,119.9 ± 5,004.3 [4,284.3–7,955.5]	*t* = −1.99 *p* = 0.05
CXCL8 ratio^*^ [pg/mL]	3.55 ± 6.53 [1.54–5.56]	0.87 ± 0.93 [0.49–1.26]	*z* = −2.080 *p* = 0.038
IL−10 non–stimulated [pg/mL]	142.3 ± 312.6 [56.1–228.4]	179.1 ± 326.5 [59.3–298.8]	*z* = 0.80 *p* = 0.42
IL−10 PHA–stimulated [pg/mL]	910.9 ± 861.9 [673.3–1148.5]	1,034.0 ± 726.5 [767.5–1,300.5]	*t* = 1.05 *p* = 0.30
IL−10 ratio^*^ [pg/mL]	962.68 ± 2,007.24 [344.95–1,580.43]	289.69 ± 703.62 [−0.75–580.12]	*z* = −1.04 *p* = 0.30

*PHA, phytohemagglutinin*.

* *PHA–stimulated to non–stimulated*.

*Data given as mean ± standard deviation [95% CI]. Bold values are statistically significant*.

### Correlations With Metabolic Parameters

Table 5 shows detailed analysis of correlations, with only significant correlations being shown. No correlations were observed for fractalkine, most correlations were between cytokines and did not include clinical or cardio-metabolic variables.

**Table 5:** Correlations in the study groups.

		**All study subjects (*n* = 92)**	**Schizophrenia (*n* = 60)**	**Control (*n* = 32)**
CXCL8 serum	IL−10 serum	rho = 0.31 *p* = 0.037	rho = 0.39 *p* = 0.033	
	IL−10 PHA–		rho = −0.55 *p* = 0.004	rho = −0.60 *p* = 0.005
CXCL8 PHA–	Age	rho = 0.24 *p* = 0.027	rho = 0.28 *p* = 0.043	
	CDSS score		rho = 0.28 *p* = 0.037	
	IL−10 PHA–	rho = 0.26 *p* = 0.047		
	CXCL8 ratio^*^	rho = −0.79 *p* <0.001	rho = −0.79 *p* <0.001	rho = −0.80 *p* <0.001
CXCL8 PHA+	IL−10 PHA–	rho = −0.48 *p* <0.001	rho = −0.49 *p* <0.001	
	IL−10 PHA+	rho = −0.60 *p* <0.001	rho = −0.74 *p* <0.001	
	CXCL8 ratio^*^	rho = 0.40 *p* <0.001	rho = 0.56 *p* <0.001	
IL−10 PHA–	IL−10 PHA+	rho = 0.38 *p* = 0.001	rho = 0.46 *p* = 0.002	
	IL−10 ratio^*^	rho = −0.95 *p* <0.001	rho = −0.96 *p* <0.001	rho = −0.93 *p* <0.001
IL−10 PHA+	CXCL8 ratio^*^		rho = −0.35 *p* <0.001	

*PHA+, phytohemagglutinin-stimulated PBMCs; PHA–, non-stimulated PBMCs; CDSS, Calgary Depression Scale for Schizophrenia; rho, Spearman's rank correlation coefficients (with Bonferroni-adjusted significance level)*.

* *PHA+ to PHA– ratio*.

*Only statistically significant correlations are given*.

In order to exclude the potential impact of clinical variables that differed between both study groups (see Table 1), a series of adjusted means analyses was run. In each model an inter-group comparison for each cytokine was adjusted for smoking, FMI, WHR, VAT area, total fat, or VAT mass. For serum CXCL8 and IL-10 these adjustments did not influence previous results, i.e., serum CXCL8 remained higher in the schizophrenia groups (*p* < 0.05 for all comparisons), and no differences were observed for serum IL-10 (*p* > 0.05 for all comparisons). The serum fractalkine level remained significantly higher in the schizophrenia groups after adjusting for WHR, but not for other variables; still, it remained numerically lower, but not statistically significant in the schizophrenia groups for all adjustments. Differences for *in vitro* non- and PHA-stimulated CXCL8 synthesis became significant after adjusting for: FMI, VAT area, total fat, and VAT mass.

### Associations Between Cytokines and Clinical Variables—Medications

**Figure 3:**
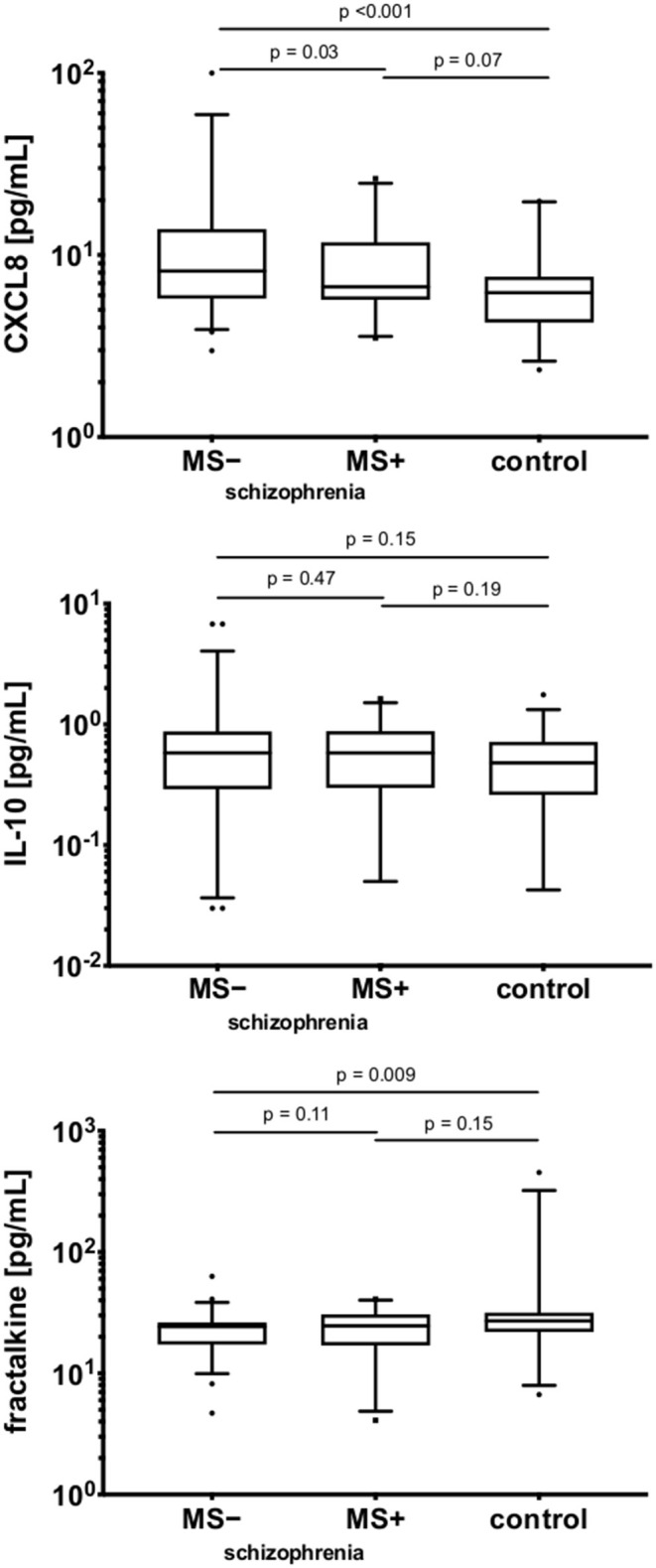
The comparison of serum CXCL8, IL-10, and fractalkine in the schizophrenia and control groups.MS −, patients on mood-stabilizers; MS +, patients without stabilizers. Y axes are log10 scaled. The boxes extend from 25 to 75th percentiles, the lines in the middle of the boxes represent medians. The whiskers extend from 10 to 90th percentiles.

**Figure 4:**
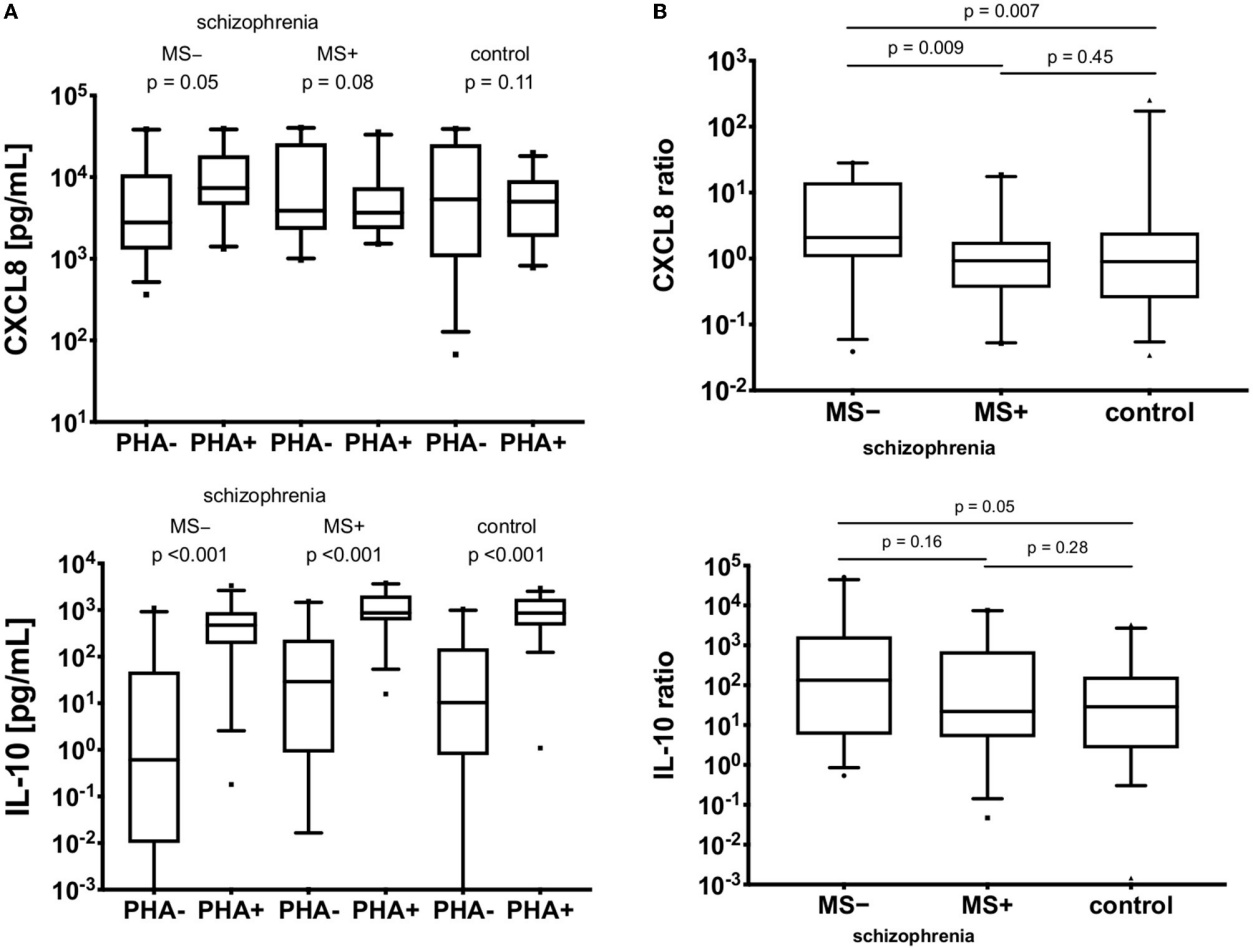
**(A)** *In vitro* CXCL8 and IL-10 production in non-PHA (PHA–) and PHA-stimulated (PHA+) PBMCs. MS−, patients on mood-stabilizers; MS+, patients without stabilizers. Vertical lines represent 95% CI, black boxes represent means. **(B)** Ratios of *in vitro* CXCL8 and IL-10 production in non-PHA (PHA–) and PHA-stimulated (PHA+) PBMCs. MS−, patients on mood-stabilizers; MS+, patients without stabilizers. The boxes extend from 25 to 75th percentiles, the lines in the middle of the boxes represent medians. The whiskers extend from 10 to 90th percentiles.

Regarding the relationships between cytokines and clinical variables, no differences in cytokine expression were found to be associated with the following variables: duration, number of episodes, type and doses of antipsychotic treatment, severity or profile (positive, negative, depressive) of clinical symptoms. A significant difference was observed between schizophrenia patients on mood stabilizers (MS+) and patients not taking them (MS–), as indicated in Figure 3. The highest CXCL8 level was observed in the MS– sub-group (*p* = 0.03), however, between MS+ and control groups (*p* = 0.07) the difference was not statistically significant. Serum fractalkine was significantly lower in the MS– sub-group compared with the control group (*p* = 0.009), with no significant difference for MS+ sub-group (*p* > 0.05). For serum IL-10 no differences were observed between all three sub-groups (*p* > 0.05). PHA-stimulated to non-stimulated ratio for CXCL8 (but not for IL-10) was higher in the MS- sub-group [CXCL8: MS+: 2.22 ± 4.33 (0.30–4.13), MS–: 7.36 ± 9.61 (3.83–10.88), *z* = 2.51, *p* = 0.01; IL-10: MS+: 892.97 ± 1,988.10 (−11.99–1,797.95), MS–: 4,124.26 ± 11,321.92 (−656.56–8,905.09), *z* = 0.91 *p* = 0.37]. However, further analysis of the differences in cytokines profiles between MS+ and MS– subjects (Figure 4A) revealed a difference in *in vitro* CXCL8 ratio between MS+, MS– patients and healthy controls (Figure 4B).

## Discussion

In the present study we have evaluated not only serum levels of cytokines but also the *in vitro* capability of PBMCs to take part in spontaneous and PHA-stimulated cytokine production in individuals with schizophrenia. Increased CXCL8 and reduced fractalkine serum concentrations were recorded in patients with schizophrenia. Fractalkine could not be detected in PBMCs culture supernatants. IL-10 concentrations did not differ between the study groups. Although some differences in metabolic variables were observed between study groups, none were observed for CXCL8, IL-10, or fractalkine levels. In addition, IL-10 (but not CXCL8) levels differed between non- and PHA-stimulated PBMCs. However, induced secretion of CXCL8 (but not IL-10), i.e., the ratio of PHA-stimulated to non-stimulated secretion, increased significantly in the schizophrenia group. It is well-known that the circulating cytokines found in serum or plasma can be produced by a broad range of cells not only immunocompetent cells but also endothelial and epithelial cells, adipocytes and fibroblasts. This knowledge may explain the observed differences between the serum levels of the analyzed cytokines and their levels released *in vitro* by PBMCs. Moreover, it appears that measurement of *in vitro* secretion of cytokines/chemokines by PBMCs may better reflect the potential of cytokines to influence inflammation.

Additionally, mood stabilizers (lamotrigine, valproate) appeared to have a potential effect on the serum concentration of CXCL8 and fractalkine, but not IL-10. Serum CXCL8 levels were significantly higher in MS- patients than in MS+ patients and control group. While serum fractalkine levels differed between MS– patients and controls, no difference was found between MS- and schizophrenia MS+ patients.

These effects could be accounted for pseudo-normalization of CXCL8 and fractalkine in schizophrenia patients taking mood stabilizers. However, the treatment with mood stabilizers did not affect IL-10 levels after PHA stimulation. For CXCL8, MS– patients had the higher increase in PHA-stimulated production.

IL-10 has been found to induce CX3CR1 expression on monocytes (26), no differences in IL-10 levels were found between the groups in the present study.

In addition, it was not possible to measure the fractalkine produced by PBMCs. It is now known that in the brain fractalkine is expressed mainly by neurons, and it's its receptor, CX3CR1, is present on microglia (19). There are data that this unique receptor is expressed on monocytes (27), subsets of T lymphocytes (28), natural killer cells (29), and dendritic cells (30). To date, there is insufficient data on the release of this chemokine by subpopulations of immunocompetent cells. In this study, we set out to perform an *in vitro* assessment of fractalkine release by peripheral blood mononuclear cells with or without stimulation to confirm that this chemokine is not produced and released by peripheral immune cells. In addition to the key source of brain-derived fractalkine, there are data that fractalkine may be proteolytically released from the cell surface by different proteases. Thus, proteolytical release of fractalkine from endothelial cells might be also a rich source of fractalkine in the circulation (31).

Serum fractalkine levels in schizophrenia might be lowered due to dysfunction in neurogenesis of CNS, rather than due to changes in anti-inflammatory components (32). Its low level in schizophrenia may contribute to neuronal pathophysiology of this condition. Hill et al. (33) reported lowered levels of this fraktalkine proteins in the brains of schizophrenia patients post mortem (33). Fractalkine interacts with the CX3 chemokine receptor 1 (CX3CR1), which is present in microglia (29, 34). CX3CL1 promotes microglial and neuronal activation, which enables the local modulation of inflammatory activity in the CNS (35). In mice, fractalkine is expressed at high levels by neurons in olfactory bulb, cortex, striatum and hippocampus in physiological conditions, with almost no expression in the cerebellum or substantia nigra (36).

The pro-inflammatory cytokines (IL-1β, IFN-γ, and TNF-α) regulate the local synthesis and expression of CX3CL1. Those cytokines, together with other factors, influence CX3CL1 production through intracellular messengers and transcription factors (37).

Regarding other neuropsychiatric disorders, the CX3CL1/CX3CR1 axis was shown to contribute to the pathology of Alzheimer's disease (AD) by altering the levels of Tau protein and the process of Tau phagocytosis in microglia cells (38). Kim et al. (39) found soluble CX3CL1 plasma expression to significantly increase with the severity of AD. Studies using animal models have also indicated a link between CX3CL1/CX3CR1 and severity of depressive behavior and cognitive impairment (40). In addition, the fracktalkine receptor was found to be physiologically responsible for cognitive functions and synaptic plasticity, CX3CR1-deficient mice demonstrated impaired motor learning, cognitive functioning and reduced hippocampal-dependent long-term potentiation (41). Zhou et al. (42) reported a link between microglial activation, CXCR1 and schizophrenia-related behaviors induced by social isolation in mice (42).

Our findings on CXCL8 levels are consistent with the majority of studies, which found its serum levels to be significantly increased in comparison with healthy controls (16, 17). Increased levels of CXCL8 have also been associated with the clinical severity of schizophrenia measured by the PANSS scale (43). Studies on IL-10 are more inconclusive. While Ajami et al. (44) report no significant differences in serum IL-10 levels between schizophrenia patients before treatment and the healthy group, as noted in the present study (44). Maes et al. (16) showed increased IL-10 serum levels in schizophrenia patients comparing to control group and Xiu et al. report decreased levels in non-treated patients (22).

Studies suggest that antipsychotics may have anti-inflammatory effects (45). Such effects have been observed for lithium in the treatment of bipolar disorder (46). The anti-inflammatory potential of lithium may result from inhibition of glycogen synthase kinase-3β (GSK-3β) which further regulates cytokines production, although other mechanisms are also considered to contribute (47). However, little is known about the effects of other mood stabilizers, such as valproate or lamotrigine on cytokine levels. Their immunosuppressive effect as well as occasional immunostimulating one has been observed in (48). It has been suggested that valproic acid may modulate the immune response by affecting dendritic cell differentiation and functions (49). Another study showed *in vitro* significant reduction of IL-10 and CXCL8 levels after treatment with valproic acid in human macrophages infected with the Dengue Virus (50). In addition, promising results were observed in a study showing an anti-inflammatory effect after intraperitoneal injection of valproic acid in rats with neuropathic pain (51). Additionally another study showed that lamotrigine was found to modulate immune response on rat models (52). The immunosuppressive effect on cytokines maybe the cause of pseudonormalization of those parameters observed in our study. Similar observation applies to antipsychotic treatment (53). The results are insufficient to determine whether the immunomodulating effect of antipsychotic and mood stabilizing medication may be compared.

While some clinical or cardio-metabolic parameters differed between the study groups the potential confounding effects for many of these variables were excluded. Nethertheless, our study does have some potential limitations. The study has a prospective, cross-sectional design, which limits the potential to infer causative effects. A small number of control subjects were included, which might have reduced the statistical power of detecting potential differences. Neither patients nor controls were not matched for sex or age. In addition, the heterogenous treatment regimens received by the patients might also have affected the observed results; although with the exception of mood stabilizers, no antipsychotic or antidepressive medications appeared to have any significant effects. Another limitation is the lack of determination of the level of the investigated cytokines in the CSF. Nevertheless, this should be definitely taken into consideration when interpreting our findings.

In conclusion cytokines may be associated with the etiopathology of schizophrenia, although the mechanism of this observation is still unclear. Our findings underline the need to consider other aspects of illness, such as medication, as potential confounding factors in this area of research.

## Data Availability Statement

The original contributions presented in the study are included in the article/supplementary material, further inquiries can be directed to the corresponding author.

## Ethics Statement

This study was performed in line with the principles of the Declaration of Helsinki. Approval was granted by the Ethics Committee of Medical University of Lodz RNN/122/16/KE. The patients/participants provided their written informed consent to participate in this study.

## Author Contributions

All authors contributed to the study conception and design. Material preparation, data collection, and analysis were performed by AW, EB-B, and EK. The first draft of the manuscript was written by JA. All authors commented on previous versions of the manuscript. All authors read and approved the final manuscript.

## Conflict of Interest

The authors declare that the research was conducted in the absence of any commercial or financial relationships that could be construed as a potential conflict of interest.

## Publisher's Note

All claims expressed in this article are solely those of the authors and do not necessarily represent those of their affiliated organizations, or those of the publisher, the editors and the reviewers. Any product that may be evaluated in this article, or claim that may be made by its manufacturer, is not guaranteed or endorsed by the publisher.
